# Geographic parthenogenesis and plant-enemy interactions in the common dandelion

**DOI:** 10.1186/1471-2148-13-23

**Published:** 2013-01-28

**Authors:** Koen JF Verhoeven, Arjen Biere

**Affiliations:** 1 Department of Terrestrial Ecology, Netherlands Institute of Ecology (NIOO-KNAW), Droevendaalsesteeg 10, Wageningen, 6708 PB, The Netherlands

**Keywords:** Red Queen, *Taraxacum*, Plant-insect interactions, Plant-pathogen interactions, Soil feedback

## Abstract

**Background:**

Many species with sexual and asexual variants show a pattern of geographic parthenogenesis where asexuals have broader and higher-latitude distribution than sexuals. Because sexual reproduction is often considered a costly evolutionary strategy that is advantageous in the face of selection by coevolving pests and pathogens, one possible explanation for geographic parthenogenesis is that populations at higher latitudes are exposed to fewer pests and pathogens. We tested this hypothesis in the common dandelion (*Taraxacum officinale*), a species with well-established geographic parthenogenesis, by screening prevalence and effects of several specialized pests and pathogens in natural dandelion populations.

**Results:**

We did a population survey of 18 dandelion populations along a geographic transect that ranged from the area where sexual and asexual dandelions co-occur northward into the area where only asexuals occur. In addition we used four southern and four northern populations in a 8x8 cross-inoculation greenhouse experiment in which plants were exposed experimentally to each other’s natural field soil microbial communities. The cross-inoculation experiment indicated a higher pathogenicity of soil microbial communities from the southern, mostly sexual, populations compared to soil microbial communities from the northern asexual populations. Northern dandelion populations also showed reduced infestation by a specialized seed-eating weevil. A similar trend of reduced rust fungus infection in northern populations was observed but this trend was not statistically significant.

**Conclusions:**

The prevalence of pests and pathogens decreased along the south-to-north axis of geographic parthenogenesis. This highlights the potential of biotic interactions in shaping patterns of geographic parthenogenesis.

## Background

Geographic parthenogenesis, or the broader and higher-latitude geographic distribution of asexuals compared to their sexual counterparts in taxa where both types coexist, is well-established in both plants and animals [1-5]. Several non-exclusive explanations have been proposed to account for this pattern [6-10]. Popular hypotheses include higher phenotypic plasticity in asexuals due to efficient selection for general-purpose genotypes [11]; better colonizing abilities of asexuals that facilitate range expansion e.g. into previously glaciated areas [1,12]; and reduced pressure from pests and pathogens at higher latitudes that allows asexuals to capitalize on their reproductive advantage compared to sexuals [13,14].

The latter ‘biotic interactions’ hypothesis rests on two assumptions. First, it builds on the idea that sexual reproduction is inherently costly compared to asexual reproduction but is advantageous in the face of specialized coevolving pests and pathogens, presumably because sex generates genetically variable offspring which reduces the risk of infection. This Red Queen (RQ) hypothesis for the evolutionary maintenance of sexual reproduction [13,15,16] has received empirical support in several systems and ecological studies have confirmed key assumptions and predictions of the hypothesis [17-20]. Still, as modeling studies show [21], it remains under debate how general the RQ hypothesis is in explaining host sexual reproduction and many alternative hypotheses for the maintenance of sexual reproduction exist [22].

The second assumption is that herbivory and pathogen pressure is less intense at higher latitudes. This has been a commonly accepted idea with considerable empirical support [23-25]. Recently, however, the existence of a latitudinal trend in plant herbivory and associated plant defenses was challenged by meta-analysis [26] and large-scale empirical screening, which both failed to support the idea [27]. However, as argued by Johnson and Rasmann [28], the patterns predicted by Coley and Aide [25] may be more readily observed by comparing geographical trends in species with similar life history and roughly comparable habitats than in the type of meta-analyses conducted by Moles et al. [26], which include a wide variety of plant growth forms, habitats, and sections of continuous latitudinal ranges. Plant-pathogen interactions have been less studied than plant-herbivore interactions in a latitudinal context [24] and relatively little empirical evidence exists to support the idea that plant pathogen pressure is lower at higher latitudes. These studies have typically been performed either across small latitudinal ranges, spanning less than one [29] up to fifteen degrees latitude [30-32], or on wider geographic scales but focusing on microbial diversity in general rather than on pathogen pressure [33]. If a pattern of decreasing herbivory and pathogen pressure with increasing latitude is in fact absent then the biotic interactions hypothesis cannot be a general explanation for geographic parthenogenesis.

In plants, little empirical support exists that geographic parthenogenesis is correlated with relaxed pest and pathogen pressure in high-latitude asexual populations compared to low-latitude sexual populations [34-36]. Here, we test for this pattern in the common dandelion (*Taraxacum officinale* sensu lato, Asteraceae), a species with well-established geographic parthenogenesis [37-39], by linking dandelion sex-asex variation to geographic variation in the occurrence of several specialized dandelion pathogens and herbivores. Sexual *T. officinale* are diploid self-incompatible plants whereas asexual *T. officinale* are triploid obligate apomicts; both forms co-occur in south-central Europe but only the asexuals extend much further northward into Scandinavia. First, we experimentally evaluated pathogenic effects of natural soil microbial communities by comparing pathogenicity between soils from southern (predominantly sexual) populations and soils from northern (asexual) populations. Soil pathogens are little explored in a RQ context but they play important roles in shaping plant communities [40]. Second, we surveyed natural dandelion populations along the south–north axis of geographic parthenogenesis to record field prevalence of infection by a specialized rust fungus and a specialized seed-eating weevil. Infection prevalence does not necessarily reflect pathogen pressure and the risk of infection, because prevalence also depends on host susceptibility which may differ between populations. However, prevalence is often used as an initial proxy for population-level differences in infection risk, and this can be a reasonable proxy especially when between-population differences in prevalence are large [41].

The overall hypothesis that we test is that the asexual dandelion populations in the northern part of the transect are exposed to lower herbivore and pathogen pressure compared to the mostly sexual dandelion populations from the southern part of the transect. This tests a key assumption of the hypothesis that antagonists are causally responsible for dandelion geographic parthenogenesis.

## Results

### Sex-asex variation between dandelion populations

Ploidy analysis confirmed the previously established pattern of geographic parthenogenesis in this species. Populations from the northern part of the transect (Sweden, Denmark, Netherlands, Belgium) were all triploid and thus apomictic. The southern part (Switzerland, eastern France, southern Germany) contained apomictic, sexual and mixed populations (Table 1).

**Table 1:** **Characteristics and infection proportions of the*T. officinale*populations**

**Site**	**Latitude**	**Longitude**	**Elevation (m)**	**% Apomicts**	**Rust infection**	**Weevil infection**
South:						
F1	46.608	3.826	256	18	0.48	n.d.
F2	46.591	4.510	301	15	0	0.17
F3	46.660	5.723	540	0	0	0.33
F4	47.295	6.803	554	0	0.54	0.35
F5	47.989	4.933	251	100	0.05	n.d.
F6	48.319	5.704	302	48	0.03	n.d.
F7	48.443	7.190	366	100	0.40	0.34
S1	47.154	7.009	789	0	0	0.73
G1	47.726	7.874	459	0	0.81	0.46
G2	48.202	8.117	421	0	0.27	0.18
North:						
B1	50.547	5.822	226	100	0	0.01
B2	50.976	5.7250	34	100	0	n.d.
N1	50.838	5.853	117	100	0.05	0.08
N2	51.958	5.742	8	100	0.49	n.d.
D1	55.379	9.228	39	100	0	n.d.
D2	55.570	11.896	29	100	0	0.37
D3	55.666	11.839	29	100	0	0.19
Sw1	55.686	13.482	30	100	0	0.01

See Figure 3 for population locations. Infection rates are the proportion of infected individuals within the population sample. Weevil infection was not determined (n.d.) in some populations.

### Soil inoculation effects on plant growth

**Figure 1:**
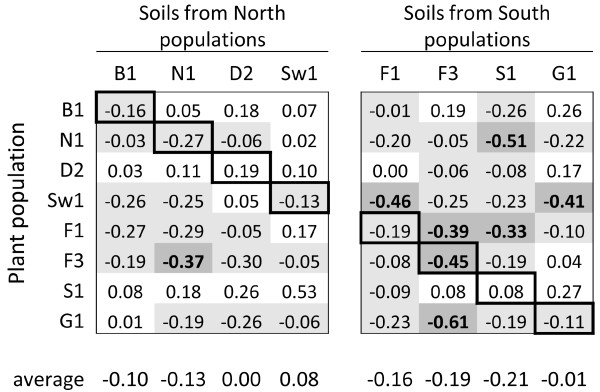
**Population-level effects of field soil inocula on plant growth.** Shown are log_2_ response ratios of shoot biomass obtained in inoculated soils relative to control soils, based on least squares means for each population x soil combination as obtained from the statistical model. Negative values indicate growth suppression compared to control soil and positive values indicate growth stimulation. Highlighted are suppressive effects (grey cells) and strong suppressive effects (dark grey cells, log ratio < −0.3). Highlighted diagonal cells represent plants growing in soil from their own home site.

Effects of the soil inocula, measured across plants from all eight populations and expressed as the log ratio of biomass in inoculated soil compared to control soil (log_2_[inoculated soil/control soil]), ranged from −0.01 to −0.21 for the southern soils (from predominantly sexual populations) compared to +0.08 to −0.13 for the northern soils (from apomictic populations; Figure 1). Soils from the southern sites tended to cause stronger plant growth suppression than soils from the northern sites (contrast test ‘south versus north soils’, F_1,110_ = 3.9, p = 0.051, see Table 2). Individual soils showed large variation in effects on different plant populations, with strong growth suppression effects observed more often in soils from southern populations. A significant effect of leaf length indicated, as expected, that plants that are larger at the start of the soil inoculation treatment have higher final biomass; by including this factor as a covariate in the statistical model our test for soil inoculum effects is independent of size differences between individuals at the start of the treatment. There was no evidence that plant populations showed a different growth response to soils from their local home sites compared to soils from other sites (Figure 1, no distinct pattern in the effects observed in diagonal versus off-diagonal cells; contrast test ‘own versus foreign soils’, F_8,774_ = 1.5, p = 0.15).

**Table 2:** Split-plot analysis of the soil feedback experiment, testing for soil effects on plant shoot biomass

**Factor (d.f.)**	**Error term**	**F**	**p**
Pre-treatment leaf length (1)	Model residual	687	<0.001
Block (2)	Model residual	41.9	<0.001
Soil (7)	Soil x Rep(Block)	2.0	0.061
*contrast ‘south vs. north soils’ (1)*	Soil x Rep(Block)	3.9	0.051
Soil x Replicate(Block) (110)	Model residual	2.6	<0.001
Plant population (7)	Model residual	8.9	<0.001
Soil x Plant population (49)	Model residual	1.2	0.131

Degrees of freedom are given in parentheses, the model residual has 774 degrees of freedom. The ‘south versus north soils’ contrast tests whether shoot biomass differs when plants are exposed to soils from the four southern (predominantly sexual) populations compared to soils from the four northern (apomictic) populations.

**Figure 2:**
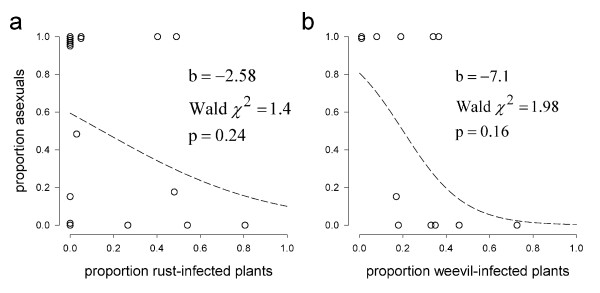
**Population-level association between the proportion of asexual plants and infection prevalence. a**. Association with incidence of rust infection (18 populations). **b**. Association with weevil larvae infection (12 populations). Dashed lines are logistic regressions based on population proportions. Overlapping data points at x,y = 0,0 or 0,1 are jittered for the purpose of visualization.

To test whether prevalence of a specialized rust fungus and the specialized *Taraxacum* weevil *Glocianus punctiger* differ along the north–south transect we compared the proportions of infected plants per population between the northern part (8 populations) and the southern part of the transect (10 populations, see Table 1). Northern populations showed lower levels of weevil infection (mean proportion of weevil-infected plants in northern populations: 0.13; in southern populations: 0.37; *t*-test of north–south comparison: *t* = −2.29, df = 10, p = 0.045). Rust infection also tended to be lower in northern populations (mean proportion of rust-infected plants in northern populations: 0.07; in southern populations: 0.26) but this difference was not statistically significant (*t*-test of north–south comparison: *t* = −1.64, df = 16, p = 0.12). Consistent with the observed north–south patterns in rust and weevil incidence, infection levels by rust and weevils tended to be lower in apomictic populations than in sexual populations; however, these relations were not statistically significant (Figure 2; logistic regression parameters and test statistics from PROC GENMOD in SAS 9.1).

## Discussion

The biotic interactions (RQ) hypothesis for geographic parthenogenesis assumes, first, that herbivory and pathogen pressure is less intense at higher latitudes; and second that pressure from coevolving pests and pathogens selects for host sexual reproduction while absence of this pressure selects for asexual reproduction. Our study tested and tentatively confirmed the first assumption and is further consistent with the second assumption. For all three types of antagonists that we tested, northern dandelion populations on average showed reduced antagonist prevalence or impact compared to southern dandelion populations, and this was generally correlated to dandelion sex-asex variation and geographic parthenogenesis. The statistical support for this pattern varied among antagonists, being significant for weevils, very close to significant for soil pathogens (p = 0.051) and non-significant for rust incidence. However, the consistency of the relationship in all antagonist types lends some confidence to the observed pattern. The lack of statistical significance may be a statistical power issue due to a comparatively limited number of populations included in our study, relative to large inter-population variation. For instance, similar surveys in other systems demonstrated the same pattern using larger numbers of populations [42,43]. A positive correlation between pest/pathogen prevalence and host sexual reproduction has been found in some but not in all systems that have been comprehensively studied [44,45].

Recent studies have challenged the existence of a latitudinal trend in herbivory [26,27]. Large scale studies of latitudinal trends in herbivory often compare different species and systems. It has been pointed out that better control for phylogenetic differences should provide better tests of the latitudinal trends [27,46]. Studies such as ours, which compare populations of the same species along a latitudinal transect, may provide the best possible test cases for the existence of a latitudinal trend in plant-antagonist interactions because the patterns are not confounded by species or community-specific differences along the latitudinal transect.

Our results are consistent with, but do not directly confirm, the assumption that antagonists select for host sexual reproduction. Better support for this hypothesis could come from observing the same correlation between antagonists and host sex-asex variation in randomly selected populations, not in populations sampled along the north–south transect of geographic parthenogenesis (because of possible confounding effects of other factors that might cause geographic parthenogenesis). Population surveys in different species have previously explored a correlation between host sex-asex variation and antagonist prevalence, confirming RQ predictions in some systems [42,43,47] but not in others [44,45,48]. If we look at the populations only in the southern part of the dandelion transect, where sexuals and asexuals coexist, the association between infection rate and the proportion of asexuals also showed a negative trend across populations (β = −0.16 for the asexual-rust association, n = 10 population; β = −0.26 for the asexual-weevil association, n = 7 populations). This conforms to the RQ predictions, however, the trends are not significant and the low number of populations makes it difficult to confidently interpret this result. In general, testing RQ predictions in areas of mixed sex-asex dandelion populations may be complicated by potentially high levels of clonal diversity within apomictic dandelion populations [49]. Because pests and pathogens may select for host diversity rather than host sexual reproduction per se, sexual reproduction is not necessarily the winning strategy under high pest and pathogen pressure when competing with a diverse population of asexual genotypes [50].

Our study provides a first evaluation of the patterns of incidence for some of the antagonists that are relevant in natural dandelion populations. Of the tested antagonists, the rust fungus may be the most relevant in a RQ context. Our survey shows that it is widespread and often infects a large proportion of plants within a population. Moreover, rusts can have severe fitness impacts in natural plant populations [51,52] and can track common host genotypes and potentially drive negative frequency dependent selection within populations [53,54]. Soil pathogens have not previously been considered in plant RQ studies. We show that pathogenic effects of soil communities are common and can result in dandelion growth reduction of 20% or more (log_2_ response ratio ≤ 0.3). This is the net effect of all soil microbes present in the soil communities. Identifying fitness effects of individual soil pathogen species may not be straightforward, but the widespread occurrence of soil pathogenic effects highlights the impact of soil antagonists for dandelion performance. We detected the weevil *G. punctiger* in all populations along the transect that we screened for weevil larvae presence. This widespread occurrence, combined with generally high infection rates within populations (Table 1) and high levels of seed predation within infected capitula [55], suggest that *G. punctiger* is a dominant herbivore in dandelion. However, it is unknown whether the dandelion-weevil interaction shows enough genotype-specificity to play a role in RQ dynamics.

In species with geographic parthenogenesis, including *T. officinale*, the asexual variant often has an increased ploidy level compared to the sexual variant. It has been argued that ploidy differences, not reproductive mode, can be responsible for patterns of geographic parthenogenesis [1,6]. However, where sex-asex variation exists in plants without ploidy level change, a pattern of geographic parthenogenesis can still be observed [34]. In *Boechera holboellii*, where polyploidy and apomixis can be uncoupled and diploid and polyploid apomicts exist, diploids extend more extensively into previously glaciated areas than polyploids [56]. Although ploidy and not reproductive mode may contribute to geographic parthenogenesis in some systems [6], such observations suggest that polyploidy per se is not a general and sufficient explanation for geographic parthenogenesis.

## Conclusions

Our study suggests that dandelion populations at higher latitudes are exposed to reduced herbivores and pathogens and this pattern generally correlates with dandelion geographic parthenogenesis. This highlights the possibility that antagonists drive patterns of geographic parthenogenesis. Whether or not antagonists are in fact causal to dandelion geographic parthenogenesis depends on the validity of the RQ hypothesis for sexual reproduction, which remains to be further demonstrated in this system.

## Methods

### Dandelion antagonists

In order to drive RQ dynamics, it is thought that the antagonist’s effect on host plant fitness needs to be severe as well as genotype-specific, such that some host genotypes in the population are less susceptible than others. Because little a priori knowledge is available on which antagonists are most relevant to dandelion fitness, our approach was to monitor a range of different antagonists:

#### Soil microbial pathogens

Effects of soil microbial communities on plants can be tested experimentally using soil inoculums and soil feedback experiments. In such experiments the net effect of a ‘living’ soil (potentially containing both mutualist and antagonist microbial species) on plant growth is evaluated [57]. Many studies have indicated that soil inoculum effects, when compared to appropriate controls, are often negative, implying overall pathogenic effects of soil microbial communities [40]. Pathogen build-up in soils is plant species-specific [58-60] and plants often suffer more from the soil microbial communities that they accumulate themselves than from soil microbial communities associated with other plant species [61]. Intraspecific variation in soil feedbacks has also been demonstrated [62] which suggests that plant genotypes within a population might differ in the pathogen communities that they accumulate and/or in their vulnerability to the same pathogen communities.

#### Rust fungus

Rust fungi and their host plants show highly coevolved interactions based on gene-for-gene recognition mechanisms [63]. Such mechanisms are characterized by genotypic variation in host sensitivity and pathogen virulence. Although it is debated whether gene-for-gene systems lend themselves easily to RQ dynamics [64-66], rust fungi are considered as potential drivers of negative frequency-dependent selection and RQ dynamics in host plant populations [53,54]. A specialized rust fungus, *Puccinia hieracii* infects *Taraxacum* in a genotype-dependent way [67] and several other rust fungus species can also infect *T. officinale*[68]. Fitness impact of rust infection is unknown in *T. officinale* but is reported to be significant in related Asteraceae species [51,52]. In the present field study no attempt was made to identify the rust species.

#### Seed-eating weevil

*Glocianus punctiger* is a seed-eating weevil that specializes on *Taraxacum*[69]. It is considered a dominant pre-dispersal predator of *Taraxacum* seeds [55]. Up to 30% of seeds per capitulum can be lost to weevil predation [55] suggesting considerable fitness consequences of infestation.

### Population survey

#### Field sampling

**Figure 3:**
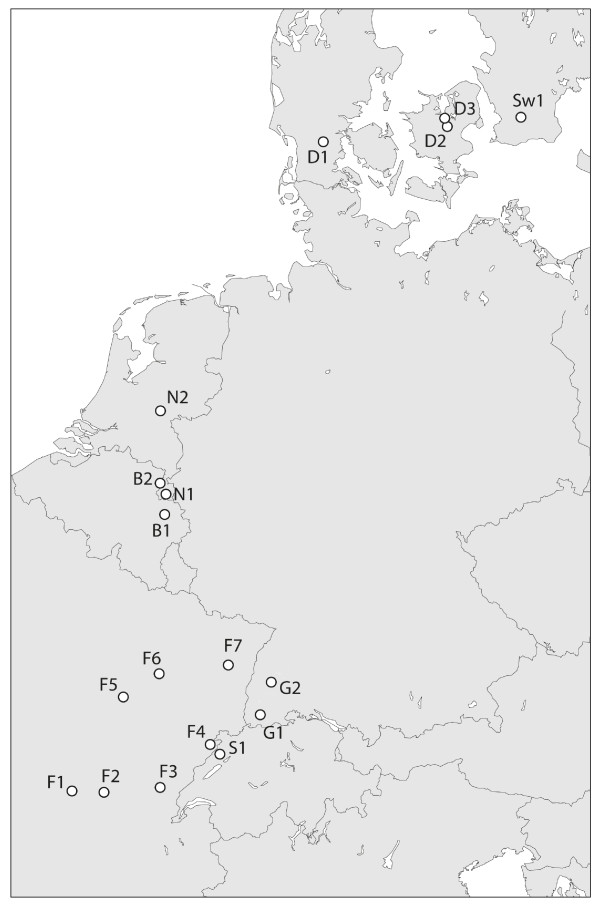
**Locations of *T. officinale *sampling sites. Labels correspond to Table **1**.**

*Taraxacum officinale* populations were sampled in hay meadows and grazed pastures from eastern France to southern Sweden during spring 2005 (18 populations in total, Figure 3). In each population 100 plants were sampled along 5 transects of 20 m, selecting the closest individual with ripe seeds at 1-meter intervals. From each plant, one capitulum with ripe seeds was collected for subsequent greenhouse propagation and ploidy analysis (see below). Each plant was checked for visible signs of leaf rust infection, usually orange-brown uredinia pustules but also larger aecia in some populations. In 12 of the populations we additionally screened for *Glocianus punctiger* infestation by randomly selecting 100 developing capitula and inspecting them for the presence of weevil larvae inside. At the growing sites of four apomictic populations from the northern part of the transect (B1, N1, D2, Sw1) and four predominantly sexual populations from the southern part of the transect (F1, F3, S1, G1; see Figure 3) field soil samples were collected from the upper 15 cm soil layer, taken at several locations per field site and subsequently mixed to yield one bulk sample per site. Soil samples were stored at 4°C until experimentation.

#### Ploidy analysis

Per population, seeds from 30 collected capitula were germinated and seedlings were grown under standard greenhouse conditions. Ploidy level was determined in leaf tissue from two seedlings from each collected capitulum, using a Partec Ploidy Analyzer ultraviolet flow cytometer to measure nuclear DNA content relative to a reference sample of known diploidy [70]. In virtually all cases the two seedlings were either both diploid or both triploid, indicating respectively sexual or apomictic status of the field-sampled mother plant [70]. In mixed populations with both diploids and triploids we screened an additional 30–70 plants to improve the estimation of the proportion of apomicts in the population.

### Soil inoculation experiment

#### Experimental design

Plants from the four northern populations (B1, N1, D2, Sw1) and the four southern populations (F1, F3, S1, G1) were exposed to field-collected soils from all eight populations in a cross-inoculation greenhouse experiment. Seeds from 15 plants per population were germinated in petri dishes on moist filter paper and seedlings were transplanted individually into 38 ml seedling tray cells filled with sterilized mineral sand (sterilized by gamma radiation ≥ 25 kGray). Eight days after transplanting, 4 ml of field soil suspension was added to each individual plant. Soil suspensions were made by adding demi water to field-collected soil samples to a final mass ratio of ¼ dry soil and ¾ water (adjusted for soil moisture content as determined by evaporative water loss in oven-dried test samples), thorough mixing (1h at 170 rpm) and passing the mixture through a 200-μm mesh sieve. 15 plants from each of the eight populations were grown with each of the eight soils, resulting in 960 plants in the experiment. An additional 30 plants per population were grown in sterilized sand without added soil suspension (control soils), in order to evaluate the overall effect of soil addition on plant growth. Seedling tray cells were placed in plastic containers in groups of eight (one plant per population) with all plants per container receiving the same soil suspension. Containers were placed on a greenhouse bench in a randomized block design, with three blocks that each contained five replicates of each population x soil treatment (blocks corresponded to three consecutive days for setting up the entire experiment). Plants received 0.5 strength Hoagland nutrient solution 1x per week and demi water 2x per week during the experiment. Plants were harvested four weeks after transplanting. Shoot biomass was determined after oven drying at 70°C for 48 hours.

#### Statistical analysis

We used a split-plot design to test for soil suspension effects on shoot biomass, with ‘soil origin’ as main-plot factor (8 levels, administered at the level of containers) and ‘plant population’ as split-plot level (8 levels, at the level of individual cells within containers). Of specific interest is the a priori contrast between soils from southern (predominantly sexual) versus northern (apomictic) populations. This explicitly tests the hypothesis that geographic parthenogenesis is correlated with differences in soil pathogenicity, specifically with reduced pathogenicity in northern apomictic populations. Blocking in the experiment (3 levels) was included in the model as a main effect. The interaction between ‘soil origin’ and ‘replicate’, where ‘replicate’ is nested within blocks, was used as error term for testing the soil origin effect. This interaction captures all variability between replicated main-plots (soil origin), which in this experiment is contained both in the differences between main-plot replicates within each block and in the soil x block interaction. At the time of soil suspension administration we measured the length of the first true leaf in each plant and included this as a covariate in the model to account for size effects prior to the soil treatment. Analysis was carried out in SAS version 9.1 for Windows (The SAS Institute, Cary, NC).

## Competing interests

The authors declare that they have no competing interests.

## Authors’ contributions

KJFV and AB conceived of the study, KJFV designed and carried out the population survey and the greenhouse soil experiment, performed the statistical analysis and drafted the manuscript. AB participated in drafting the manuscript. Both authors read and approved the final manuscript.
